# Successful open abdomen treatment for multiple ischemic duodenal perforated ulcers in dermatomyositis

**DOI:** 10.1186/1749-7922-9-48

**Published:** 2014-08-30

**Authors:** Roberta Villa, Stefano Costa, Sibilla Focchi, Carlo Corbellini, Massimo Vigorelli, Ettore Contessini Avesani

**Affiliations:** 1 Department of General and Emergency Surgery, Fondazione IRCCS Ca’ Granda Ospedale Maggiore Policlinico, Milan, Italy; 2 Faculty of Medicine, Università degli Studi di Milano, Milano, Italy

**Keywords:** Dermatomyositis, Duodenal perforations, Retroperitoneal air, Intestinal ischemia, Acute abdomen, Emergency surgery, Open abdomen

## Abstract

**Introduction:**

Dermatomyositis is an autoimmune disease characterized by proximal myopathy, cutaneous Gottron papules and heliotrope rash; intestinal involvement associated to acute vasculitis is less common but could be a life-threatening condition.

**Methods:**

A 21-year-old woman, affected by dermatomyositis, presented to our attention with a three-day story of severe abdominal pain, no bowel movement and biliary vomit. She was diagnosed with acute abdomen. A CT scan with bowel contrast demonstrated the presence of a leakage from the retroperitoneal aspect of duodenum. The surgical and clinical management in the light of literature review is presented.

**Results:**

Our first approach consisted in primary repair of the duodenal perforation with omentopexy. Post-operative course was complicated by hemorrhage. A reintervention showed a new perforation associated with multiple ischemic intestinal areas. We performed a gastroenteric anastomosis with functional exclusion of the damaged duodenum and positioning of drainages to create a biliary fistula. A nutritional enteric tube and an open abdomen vacuum-assisted closure system to monitor the fistula creation and to prevent abdominal contamination and collections were positioned. To reduce the amount of biliary leakage, a percutaneous transhepatic biliary drainage was placed, with progressive fistula flow disappearance in four months.

**Conclusions:**

In patients with dermatomyositis, when clinical findings and symptoms suggest abdominal vasculitis, it is very important to be aware of the risk of bowel and particularly duodenal perforations. Open abdomen treatment favors control of contamination by gastrointestinal contents, offers temporary abdominal closure, helps ICU care and delays definitive surgery.

## Background

Dermatomyositis (DM) is an autoimmune disease characterized by cutaneous heliotropic rash, Gottron papules and proximal myopathy associated to dysphagia, dysphonia, Raynaud phenomenon, fatigue and non-erosive inflammatory polyarthritis [1]. Vasculitis of the gastrointestinal tract is a life threatening complication, potential cause of hemorrhage and perforation [2].

We performed a literature review by searching on PubMed (keywords: dermatomyositis, acute vasculitis, ischemic perforation, bowel perforation, emergency surgery): only few cases of bowel perforation associated to dermatomyositis are described in literature, and surgical approach is not always mentioned or specified [2-19]. In literature gastroenteric vasculitic manifestations of DM are often associated to the juvenile form [20] of the disease, affecting children in 95.1% and adults in 4,9% of cases, with clinical onset before 16 years old. To our knowledge, in literature, are reported 18 articles describing 35 cases of bowel perforation and only two cases related to adult patients (Table 1) [2-19]. Major sites of perforation are the esophagus (5,5%), the stomach (2,8%), the duodenum (25%), the ileum (2,8%), the right colon (17.1%), the transverse colon (2,8%), the sigmoid colon (2,8%) and the gastrointestinal tract with no specific site description (41,2%). Reported mortality rate is 14,3%, principally due to encephalic vasculitis and septic complications.

**Table 1:** Intestinal perforation in dermatomyositis, literature review

**Author**	**N° of cases**	**Site of perforation**	**Treatment**	**Outcome**
Zarbalian Y et al. 2013 [10]	1	Right colon	Right hemicolectomy	Uneventful
Mamyrova G et al. 2007 [9]	2	Right colon	Right hemicolectomy	Uneventful
		Duodenum	Unknown	Left hemicolectomy for left colon perforation
Morita Y et al. 2007 [3]	1	Duodenum	Primary suture repair	Uneventful
Chiu SK et al. 2007 [11]	1	Duodenum	Not described	Uneventful
Chen G et al. 2005 [12]	1	Occult perforation	Exploratory laparotomy	Fatal sepsis
Wang IJ et al. 2001 [5]	1	Duodenum	Not described	Uneventful
Suwa A et al. 1997 [8]	1	Right colon	Right hemicolectomy	Uneventful
Lin W et al. 1995 [2]	1	Sigmoid colon	Total colectomy	Fatal sepsis
Ghayad E et al. 1993 [13]	1	Colon	Not described	Not described
Niizawa M et al. 1991 [7]	1	Right colon	Right hemicolectomy	Uneventful
Downey EC et al. 1988 [14]	4	Esophago-colonic	Suture, resection and drainage	Not described
Miller LC et al. 1987 [15]	10	Esophago-colonic	Not described	Fatal sepsis
Schullinger JN et al. 1985 [16]	4	Duodenum, esophagus and colon	Partial gastrectomy, drainage	Uneventful
		Duodenum	Partial gastrectomy	Uneventful
		Stomach	Partial gastrectomy	Uneventful
		Transverse colon	Colostomy	Fatal vascular cerebral complications
Magill HL et al. 1984 [4]	2	Duodenum	Not described	Not described
Thompson JW et al. 1984 [6]	1	Esophagus	Debridement and drainage	Uneventful
Kaplinsky et al. 1978 [17]	1	Duodenum	Non described	Not described
Koiunderliev et al. 1975 [18]	1	Small bowel	Segmentary resection	Uneventful
Bureau et al. 1958 [19]	1	Duodenum	Exploratory laparotomy	Fatal sepsis

We report the case of a 21-year-old patient affected by DM presenting with rapid onset acute abdomen associated to severe vasculitis and complicated duodenal perforation, and discuss the surgical and clinical management in the light of literature review.

## Case report

**Figure 1:**
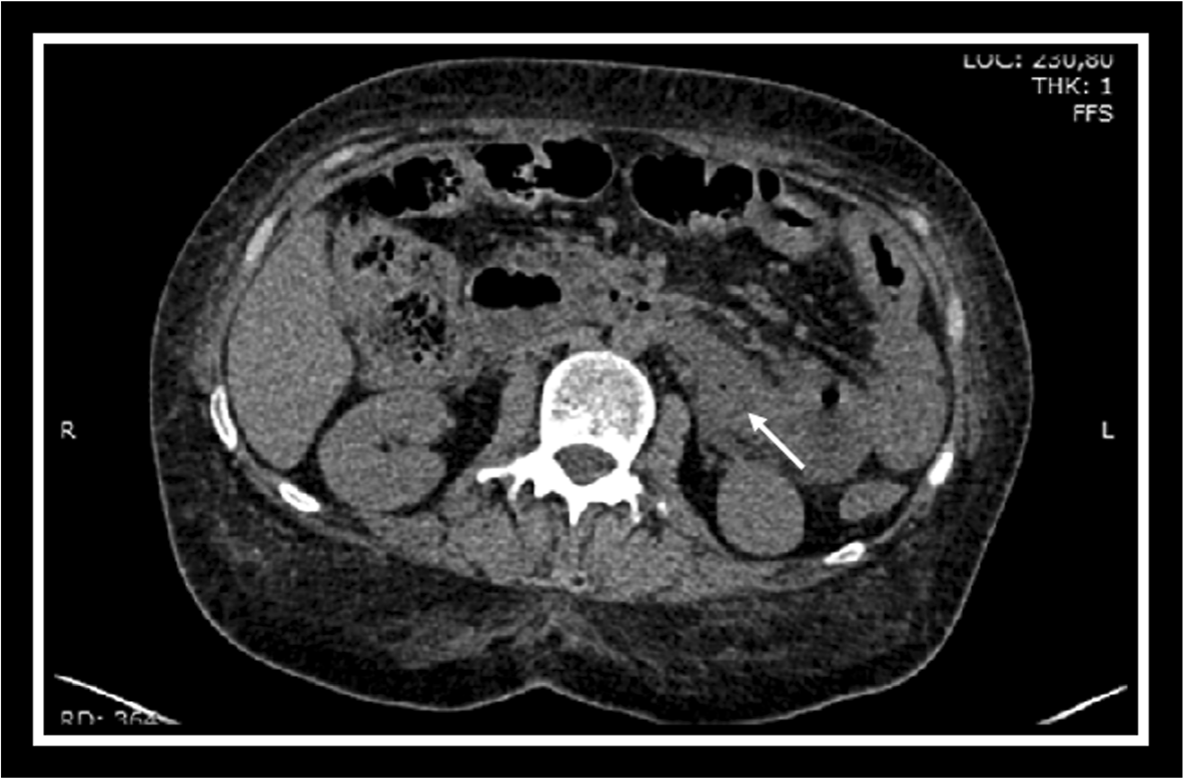
Abdominal computed tomography (CT) scan showed free retroperitoneal air (arrow), suspected for a small leakage from the posterior aspect of the third duodenal portion.

A 21-year-old female diagnosed with DM in 2008, on treatment with prednisone and cyclosporine with moderate disease activity until December 2012, presented to our Emergency Department (ED) with a three day history of diffuse, acute abdominal pain, no bowel movement and biliary vomit. She underwent laparoscopic cholecystectomy in 2010 for symptomatic calculosis. The patient was admitted to our Department with a bowel perforation suspect. An oral follow-through was negative but a CT scan with oral contrast demonstrated a small leakage from the posterior aspect of the third duodenal portion (Figure 1). An emergency laparotomy was performed, with intraoperative finding of multiple ischemic vasculitic lesions of the small bowel, retroperitoneal perforation of the third duodenal portion and a minimum local biliary contamination. The lesion was sutured with omentopexy and an abdominal drainage was placed. After surgery, the patient was transferred to Intensive Care Unit (ICU) for post-operative monitoring. Her clinical course, in the following two days, was complicated by acute hemorrhage. She underwent, therefore, a second operation due to the bleeding from a small branch of the anterior pancreaticoduodenal artery. A new ischemic perforation in the same duodenal region was recognized and multiple small ischemic areas involving the entire small bowel were observed. A gastroenteric anastomosis was performed, excluding the duodenum. Two drainages were placed near the perforated site to drain any possible biliary fistula. A nasoenteral feeding tube was then positioned. To manage the potential perforation risk of the duodenal and ileal ulcerations caused by acute vasculitis, to preserve the abdominal cavity from intraperitoneal collections and to create a guided biliary fistula, an open abdomen treatment with negative pressure system was placed; we positioned a temporary fascial mesh to preserve the fascia and prevent its retraction. Two weeks after the second surgical procedure a percutaneous transhepatic biliary drainage (PTBD) was placed to reduce the flow of the peritoneal biliary fistula.

We changed the negative pressure dressing every 3–4 days, washing the peritoneal cavity and tightening the fascial mesh. The negative pressure system was very useful and effective because of the large amount of biliary leakage and bowel contamination caused by multiple ischemic ulcers in the second and third portion of the duodenum, otherwise this condition was not manageable with the use of simple drains. After two months, the open abdomen treatment was suspended, the fascial mesh was removed and the fascia was primarily closed. Afterward, we removed the PTBD and the abdominal drain following the execution of abdominal X-ray with oral contrast, demonstrating absence of residual duodenal biliary leakage after four months.

During her ICU stay, the patient presented signs of renal vasculitis, therefore she underwent cycles of continuous veno-venous hemodialysis (CCVHD), plasmapheresis and intravenous immunoglobulin (IVIG), showing clear improvement of her renal function and negative immunological test. Low molecular weight heparin (LMWH) treatment was complicated by heparin induced thrombocytopenia (HIT) with low platelet (PLT) count (99.000/μm^3^). Argatroban was administered obtaining progressive increase in PLT count (354.000/μm^3^). Three months after surgery she had seizures with MRI scan positive for vasculitic diffuse encephalic lesions, treated with levetiracetam and metilprendisone. During hospitalization we observed nasal regurgitation of fluids, nasal speech and hoarseness probably due to loss of pharyngoesophageal muscle tone and increase and reduction in hepatic stasis values of unknown origin. After 8 months of follow-up, no signs or symptoms of abdominal disease were reported.

DM is an autoimmune disease characterized by cutaneous heliotropic rash, Gottron papules and proximal myopathy associated to dysphagia, dysphonia, Raynaud phenomenon, fatigue and non-erosive inflammatory polyarthritis [1]. Vasculitis of the gastrointestinal tract is a life threatening complication, more frequently observed in children than in adults [3]. It may vary from segmental bowel edema to ulcerations, gangrene and perforation [2]. The classic clinical findings may be masked by corticosteroids therapy and the radiographic investigations may be negative even in presence of bowel perforation, as the lesion may be very small, retroperitoneal, self sealed or well contained by the adjacent structures. Extraluminal air can be observed in 50–70% of patients [21]. Many cases involve the duodenum and particularly the third portion and its retroperitoneal aspect [3-5,9,11,16,17,19]. Other typical sites of perforation are the esophagus [6,14-16], the cecum, and the right and left colon in their retroperitoneal portion [2,7-10,13]. Histopathological findings are related to acute arteriopathy, with arterial and venous intimal hyperplasia and occlusion of vessels by fibrin thrombi. Chronic vasculopathy is characterized by reduction or complete occlusion of multiple small and medium arteries, subintimal foam cells, fibromixoid neointimal expansion and significant luminal compromise and infiltration of macrophages through the muscle layers into the intima [9,22]. In younger patients systemic vasculitis with specific involvement of renal and encephalic system can be observed. Radiological features of vasculitis include widespread thickening of mucosal fold and irregularity of small intestine, giving rise to a “stacked coin” appearance [1]. When clinical findings and symptoms suggest possible abdominal vasculitis in a young subject known for DM, it is very important to consider bowel and particularly retroperitoneal perforation. In order to manage this difficult clinical and surgical condition it is mandatory to consider the medical complexity of this disease and the necessity to treat the patient with a specific therapy to control the acute vasculitic process conditioning damage to multiple organs such as respiratory, renal and encephalic system, causing septic shock, renal failure and encephalitis. In this case, during the recovery, we had to manage gastroenteric, renal and encephalic vasculitic complications. The patient underwent three cycles of CCVHD, plasmapheresis and IVIG, multiple antibiotic coverage and careful steroid management. Her course was also complicated by heparin-induced thrombocytopenia during treatment with LMWH to prevent thromboembolism; treatment with argatroban permitted a progressive platelet count improvement. Her recovery was also complicated by dysphagia for both solids and liquids, caused by loss of pharyngoesophageal muscle tone and encephalic vasculitis, which started with seizures and was treated with levetiracetam and metilprednisolone.

Surgical treatment is not standardized because of the rarity and variety of the gastrointestinal DM presentations that can affect the entire gastrointestinal tract. In literature we found few descriptions of ischemic gastrointestinal perforation in DM. Surgical treatments and outcomes reported were anecdotic and varied, including resection of the affected tract or sutures of the lesions (Table 1). High recurrence of reintervention for anastomotic dehiscence or new perforations was observed. The use of negative pressure treatment was never reported. Open abdomen treatment allows the reduction of contamination by gastrointestinal contents decreasing the risk of abdominal collections, favors rapid evidence of hemorrhage permitting a prompt control of the bleeding source, offers temporary abdominal closure, helps ICU care and delays definitive surgery [23,24]. In this case we performed an open abdomen treatment to better remove the losses and control possible sources of new perforations, without needing of bowel resection. The mesh-mediated fascial traction technique combined with negative pressure treatment allowed to preserve the fascia, and to obtain the fascial primarily closure. As reported in literature, achievement of fascial closure has significant implications for the recovery of the patients, reducing ICU and hospital length of stay, and need for surgical reconstruction of the abdominal wall [25]. We had to perform a bowel deviation because of the critical ischemic vasculitis of the duodenum. To reduce the amount of biliary leakage and to obtain a faster outcome, we positioned a PTBD. Using this composite technique progressive fistula flow reduction was obtained, allowing abdominal closure after two months and PTBD removal after four months.

## Conclusions

When clinical findings and symptoms suggest possible abdominal vasculitis in a young subject known for DM, it is very important to consider bowel and particularly duodenal perforation. We found very helpful CT scan with oral contrast to support diagnosis and we had to face the more life-threatening condition of multiple ischemic intestinal ulcerations conditioning duodenal multiple perforations. To manage this challenging condition we used open abdomen treatment with exclusion of the duodenal ischemic perforated tract through a gastroenteroanastomosis and PTBD with the creation of a guided fistula to decrease the flow and obtaining progressive healing with improvement of patient’s general conditions. This surgical treatment must always be accompanied by DM specific medical treatment to avoid further vasculitic complications and to obtain control of the disease activity.

## Consent

Written informed consent was obtained from the patient for publication of this Case report and any accompanying images. A copy of the written consent is available for review by the Editor-in-Chief of this journal.

## Abbreviations

CAPS: Anti-phospholipidic catastrophic syndrome; CT: Computed tomography; CVVHD: Continuous veno-venous haemodialysis; DM: Dermatomyositis; ED: Emergency Department; HIT: Heparin induced thrombocytopenia; ICU: Intensive care unit; IVIG: IntraVenous immunoGlobulin; LAC: Lupus anti coaugulant; LMWH: Low molecular weight heparin; MRI: Magnetic resonance imaging; NGT: Naso-gastric tube; PTBD: Percutaneous transhepatic biliary drainage; PLT: Platelet.

## Competing interests

The authors declare that they have no competing interests.

## Authors’ contributions

RV made substantial contributions to acquisition and interpretation of data, was involved in conception and drafting of the manuscript. SC contributed to interpretation of data, was involved in conception, drafting and revision of the manuscript. SF and CC contributed to acquisition of data, drafted the manuscript. MV was involved in revising the manuscript critically for important intellectual content. ECA contributed to interpretation of data, gave final approval of the version to be published. All authors read and approved the final manuscript.
